# Confocal Laser Endomicroscopy in Brain Metastasis Surgery: A Systematic Review of the Evidence at the Tumor–Brain Interface

**DOI:** 10.3390/jcm15124420

**Published:** 2026-06-07

**Authors:** Sergio Alexander Calero Martinez, Nazeer Aboud, Paolo Ferroli, Francesco Acerbi, Morgan Broggi, Francesco Restelli

**Affiliations:** 1 Department of Neurosurgery, Fondazione IRCCS Istituto Neurologico Carlo Besta, 20133 Milan, Italy; 2 Institute of Neuroscience, Faculty of Medicine, El Bosque University, Bogota 111321, Colombia; 3 Department of Neurosurgery, University of Jena Hospital, 07747 Jena, Germany; 4 Department of Translational Research and New Technologies in Medicine and Surgery, University of Pisa, 56126 Pisa, Italy; 5 Department of Neurosurgery, Azienda Ospedaliero Universitaria Pisana, 56124 Pisa, Italy

**Keywords:** brain metastases, confocal laser endomicroscopy, sodium fluorescein, tumor margin, intraoperative imaging

## Abstract

**Background**: Brain metastases are the most common intracranial tumors in adults and are traditionally considered well-demarcated lesions amenable to complete surgical resection. Nonetheless, increasing histopathological evidence demonstrates that metastatic cells may infiltrate beyond the contrast-enhancing margin into surrounding brain parenchyma, challenging the reliability of conventional imaging for defining true tumor boundaries. Confocal laser endomicroscopy (CLE) using Sodium Fluorescein (SF) has emerged as a novel intraoperative imaging modality capable of providing real-time, high-resolution optical biopsies, potentially improving margin assessment during metastasis surgery. **Methods**: A systematic literature search was performed according to PRISMA guidelines across PubMed, Embase, Scopus, Cochrane Library, and Google Scholar up to 3 March 2026. Studies evaluating intraoperative CLE with SF in adult patients with brain metastases were included. Data regarding study design, patient population, CLE system, imaging characteristics, and diagnostic performance were extracted. Risk of bias was assessed using the QUADAS-2 tool. **Results**: Ten studies met the inclusion criteria for qualitative synthesis, comprising over 650 patients; however, most studies included heterogeneous intracranial tumor populations, with only a subset specifically involving brain metastases. CLE enabled real-time visualization of tumor microarchitecture and demonstrated high sensitivity for tumor detection, frequently exceeding 90% in prospective studies. Specificity varied across studies, reflecting challenges in distinguishing tumor infiltration from reactive tissue at the tumor–brain interface. The MetInfilt trial highlighted that infiltrative growth patterns are common in brain metastases and can be visualized intraoperatively using CLE. Additional studies demonstrated that fluorescein-based CLE allows differentiation of tumor zones and may facilitate targeted margin assessment; however, evidence demonstrating improvement in clinically meaningful outcomes such as extent of resection, local recurrence, progression-free survival, or overall survival remains limited. **Conclusions**: Confocal laser endomicroscopy using SF represents a promising intraoperative adjunct for assessing tumor margins in brain metastasis surgery. By enabling real-time microscopic visualization of the metastasis–brain interface, CLE may support a more biologically informed surgical strategy.

## 1. Introduction

Brain metastases (BM) represent the most common intracranial tumors in adults and occur in up to 20–40% of patients with systemic cancer, frequently originating from lung, breast, skin, renal, or colorectal malignancies [1] Surgical resection remains a milestone of the management of large or symptomatic BM, particularly when rapid neurological deterioration, mass effect, or diagnostic uncertainty is present [2].

Despite their traditionally circumscribed growth pattern, increasing histopathological evidence demonstrates that many BM exhibit tumor cell infiltration beyond the contrast-enhancing margins into the surrounding brain parenchyma, which may contribute to local recurrence after surgery [3,4]. The interface between metastatic tumor tissue and adjacent brain parenchyma has therefore emerged as a critical target for intraoperative assessment during metastasis removal [5,6]. Accurate intraoperative identification of tumor infiltration at this interface remains challenging because conventional surgical microscopy and neuronavigation rely primarily on macroscopic anatomical landmarks and preoperative imaging rather than real-time histological information [7].

Frozen section histopathology can provide intraoperative diagnostic confirmation but requires tissue sampling and processing [8]. Confocal laser endomicroscopy (CLE) has emerged as a promising intraoperative imaging technique capable of providing real-time, high-resolution optical biopsies of brain tissue at the cellular level during neurosurgical procedures [7]. CLE enables visualization of tissue microarchitecture through fluorescence-based imaging and allow surgeons to distinguish tumor from normal brain parenchyma without the delay associated with conventional histological processing [9]. Sodium fluorescein (SF) accumulates in areas of blood–brain barrier disruption and tumor tissue and has been widely used during fluorescence-guided neurosurgery for both primary brain tumors and metastatic lesions [10]. The administration of fluorescent contrast agents, particularly SF, enhances the diagnostic capability of CLE by enabling visualization of cellular structures, extracellular matrix patterns, and vascular architecture within tumor tissue [11].

Prospective clinical studies have demonstrated that CLE using SF can achieve high diagnostic accuracy when compared with conventional histopathology for identifying tumor tissue during brain tumor surgery [8,10]. The potential role of CLE for assessing the metastasis–brain parenchyma interface (MBPI) and detecting tumor infiltration at surgical margins remains insufficiently characterized.

Therefore, a systematic evaluation of the current clinical evidence is required to clarify the role of fluorescein-guided confocal laser endomicroscopy in metastasis surgery. The objective of this systematic review is to assess the available literature regarding the intraoperative use of confocal laser endomicroscopy with SF for identifying tumor tissue and evaluating the tumor–brain interface during brain metastasis surgery.

## 2. Methods

### 2.1. Search Strategy

A comprehensive literature search was conducted to identify all studies evaluating the intraoperative use of CLE with SF for tumor detection and margin assessment during BM surgery. The following electronic databases were systematically searched: PubMed (n = 28), Ovid (n = 0), Scopus (n = 1), Embase (n = 183), Cochrane Library (n = 1), and Google Scholar (n = 2860). The search included studies published up to 3 March 2026.

The search strategy combined Medical Subject Headings (MeSH) and free-text terms related to brain metastases, confocal laser endomicroscopy, and fluorescein. The following search query was used:

(“Brain Neoplasms” OR “Neoplasm Metastasis” OR brain metastas* OR intracranial metastas* OR metastatic brain tumor*) AND (“Microscopy, Confocal” OR “confocal laser endomicroscopy” OR “confocal endomicroscopy” OR CLE OR CONVIVO^®^) AND (“Fluorescein” OR “fluorescein sodium” OR “SF” OR fluorescein). The review protocol was not registered.

Additional articles were identified through manual screening of reference lists from relevant publications. The systematic review was conducted in accordance with the Preferred Reporting Items for Systematic Reviews and Meta-Analyses (PRISMA) guidelines.

### 2.2. Eligibility Criteria

Studies were considered eligible if they met the following criteria.

### 2.3. Inclusion Criteria

- Adult patients (≥18 years) undergoing surgical treatment for BM;
- Studies reporting the intraoperative use of CLE with SF;
- Articles providing clinical outcomes, diagnostic accuracy, or intraoperative imaging findings related to tumor tissue or tumor margins;
- Original studies including prospective studies, retrospective cohorts, case series, or case reports.

### 2.4. Exclusion Criteria

- Studies involving pediatric-only populations;
- Non-English publications without accessible full-text English translation;
- Review articles, editorials, letters, conference abstracts without full text, or expert opinions;
- Animal or preclinical studies;
- Studies using CLE without SF;
- Studies limited to ex vivo analysis without intraoperative clinical application.

### 2.5. Study Selection

All records identified through the database search were imported into a reference management software and duplicates were removed. After duplicate removal, 2676 articles remained for screening. Titles and abstracts were independently screened by two reviewers (SACM, NA). Studies that did not meet the eligibility criteria were excluded at this stage. Full texts of potentially relevant studies were then reviewed independently by three reviewers (SACM, NA, FR). A total of 33 studies were assessed for full-text eligibility. Of these, 10 studies met the inclusion criteria and were included in the qualitative systematic review. Any disagreements regarding study eligibility were resolved through discussion and consensus among the reviewers or through consultation with a fourth reviewer (MB).

**Figure 1:**
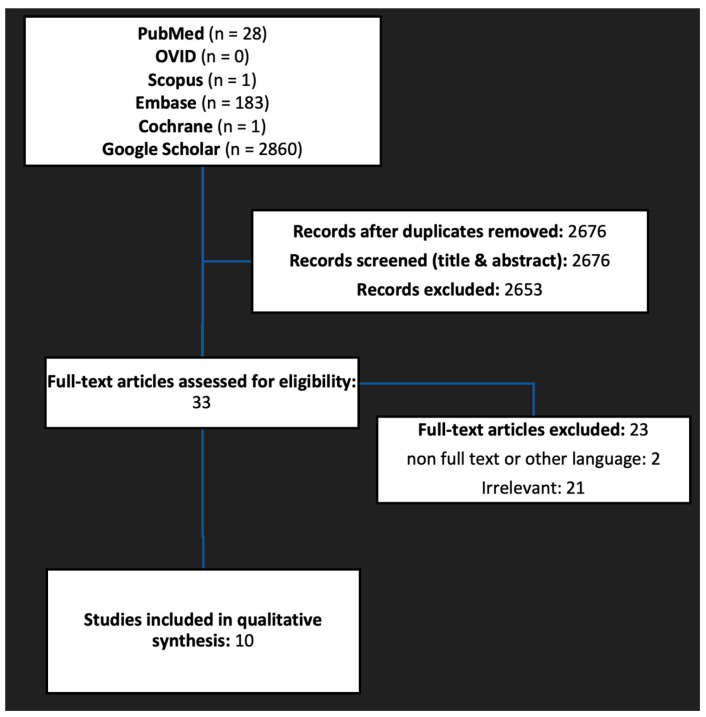
**PRISMA flow diagram of study selection.** Flow chart illustrating the study identification, screening, eligibility assessment, and final inclusion process according to the Preferred Reporting Items for Systematic Reviews and Meta-Analyses (PRISMA) guidelines. A total of 3073 records were identified through database searches. After removal of duplicates, 2676 records were screened by title and abstract. Thirty-three full-text articles were assessed for eligibility, of which 10 studies met the inclusion criteria and were included in the qualitative systematic review.

The PRISMA flow diagram summarizes the study selection process (Figure 1). The PRISMA Checklist was provided as a Supplementary document and submitted separately along with the manuscript (Supplementary Table S1).

### 2.6. Data Extraction

Data extraction was independently performed by two reviewers using a standardized data collection form. Extracted variables included:

- Study characteristics (author, year, country, study design);
- Sample size;
- Number of brain metastases included;
- CLE system used (e.g., CONVIVO^®^ (Carl Zeiss Meditech, Oberkochen, Germany) or probe-based CLE);
- Fluorescein sodium usage;
- Imaging modality (in vivo or ex vivo intraoperative imaging);
- Location of imaging (tumor core, tumor margin, tumor–brain interface);
- Reference standard used for comparison (frozen section or permanent histopathology).

### 2.7. Quality Assessment

The methodological quality and risk of bias of the included studies were assessed using the Quality Assessment of Diagnostic Accuracy Studies 2 (QUADAS-2) tool. The methodological quality of included studies is listed in Supplementary Table S2.

This tool evaluates studies across four domains:

1. Patient selection;
2. Index test (confocal laser endomicroscopy);
3. Reference standard (histopathological analysis);
4. Flow and timing between the index test and reference standard.

Each domain was assessed for risk of bias and concerns regarding applicability and categorized as *low*, *high*, or *unclear risk of bias*.

## 3. Results

### 3.1. Study Characteristics

The characteristics of the included studies are summarized in Table 1. The 10 studies were published between 2011 and 2025 and included a mixture of prospective feasibility studies, prospective clinical trials, and cohort studies. Overall, the included studies investigated the intraoperative application of CLE using SF during brain tumor surgery. Several CLE systems were used, including the ZEISS CONVIVO^®^ platform and probe-based CLE devices.

The total number of patients across all included studies exceeded 650 patients, although the number of patients with BM specifically was substantially smaller, reflecting the heterogeneous tumor populations included in many CLE studies. Among the included studies, only two studies specifically focused on brain metastases, whereas the remaining studies evaluated mixed intracranial tumor populations that included metastasis subgroups. The MetInfilt trial by Proescholdt et al. included the largest metastasis-specific cohort (50 patients) and investigated the histological characteristics of the MBPI using fluorescein-guided imaging and intraoperative CLE sampling [5]. Other studies primarily evaluated the diagnostic accuracy of CLE for differentiating tumor from normal brain tissue, comparing CLE findings with frozen section or permanent histopathology as the reference standard.

### 3.2. Intraoperative CLE Imaging and Tumor Margin Visualization

Early feasibility work by Sanai et al. demonstrated the initial clinical applicability of intraoperative confocal microscopy in human brain tumors, establishing the foundation for subsequent CLE-based intraoperative imaging approaches [15]. Across the included studies, CLE imaging enabled visualization of tumor microarchitecture at the cellular level, including tumor cell clusters, nuclear pleomorphism, and abnormal vascular structures. These features allowed differentiation between tumor tissue and surrounding brain parenchyma in real time during surgery.

Some studies have specifically described CLE imaging across different intraoperative zones, including the tumor core, tumor border, and peritumoral brain tissue. For example, Höhne et al. systematically evaluated CLE images obtained from the tumor center, tumor margin, and perilesional brain tissue, demonstrating distinct morphological patterns across these regions and highlighting the ability of CLE to assess the tumor–brain interface intraoperatively [13]. Similarly, Proescholdt et al. investigated the MBPI in a prospective cohort of brain metastasis patients and confirmed that intraoperative CLE, combined with fluorescein guidance, enables targeted sampling and visualization of this critical interface [5]. Studies focusing on metastatic tumors further suggest that SF preferentially accumulates within tumor tissue, facilitating visualization of tumor cells during CLE imaging. Brielmaier et al. demonstrated that intracellular fluorescein accumulation is significantly more frequent in metastatic brain tumors compared with primary brain tumors, which may enhance CLE-based identification of metastatic tumor cells [12]. Earlier work by Belykh et al. also showed that fluorescein-enhanced CLE imaging allows detailed visualization of tumor microarchitecture, including cellular and vascular patterns, supporting its use for intraoperative tissue characterization [10]. Collectively, these findings indicate that CLE imaging characteristics vary depending on tumor biology and anatomical location, supporting its potential role as a real-time tool for assessing tumor margins and the tumor–brain interface during brain metastasis surgery.

### 3.3. Diagnostic Accuracy of CLE

All included studies evaluated the diagnostic performance of confocal laser endomicroscopy CLE using histopathological analysis as the reference standard. However, most of the available studies evaluated CLE for general tumor detection rather than specifically assessing the metastasis–brain interface. In addition, Abramov et al. demonstrated the feasibility of telepathology-based interpretation of CLE images, highlighting the potential for remote intraoperative collaboration between surgeons and neuropathologists [9].

Early prospective work by Martirosyan et al. demonstrated that CLE using SF can achieve high diagnostic performance in brain tumor surgery, with reported sensitivity exceeding 90% for tumor detection [7]. Similarly, Abramov et al., in a prospective in vivo feasibility study using the CONVIVO^®^ system, reported high concordance between CLE findings and histopathology, with sensitivity and specificity values in the range of approximately 90% or higher [11]. Wagner et al. reported an overall diagnostic accuracy of approximately 87% when comparing CLE with frozen section histopathology, while also demonstrating a high sensitivity but comparatively lower specificity in tumor versus non-tumor classification [8]. These findings suggest that CLE may serve as a rapid intraoperative adjunct to conventional pathology, particularly for ruling in tumor tissue. Likewise, in our previous study, we reported that CLE can accurately identify tumor tissue at infiltration margins, further supporting its role in intraoperative margin assessment [14].

### 3.4. Risk of Bias Assessment

Overall, most studies demonstrated, after using the QUADAS-2 tool, a low risk of bias in the index test and reference standard domains, reflecting consistent use of histopathological analysis as the diagnostic gold standard. Nevertheless, several studies showed unclear risk of bias in patient selection and flow and timing, primarily due to small sample sizes, heterogeneous tumor populations, and incomplete reporting of inclusion criteria. Applicability concerns were generally low, although the inclusion of mixed tumor cohorts in multiple studies limits the direct generalizability of the findings to BM surgery.

## 4. Discussion

Brain metastasis has traditionally been regarded as well-demarcated lesions amenable to resection along a clear surgical plane; however, accumulating evidence indicates that this concept is overly simplistic. The prospective MetInfilt trial by Proescholdt et al. demonstrated that metastatic cells frequently extend beyond the contrast-enhancing lesion into surrounding brain parenchyma, with infiltrative growth patterns observed in most cases [5]. These findings indicate that the radiological margin does not necessarily correspond to the true biological boundary of disease and that reliance on macroscopic or imaging-based resection alone may result in residual microscopic tumor at the metastasis–brain interface.

CLE has emerged as a promising intraoperative imaging adjunct to address this limitation. By combining SF with high-resolution optical imaging, CLE enables real-time visualization of tissue microarchitecture, effectively functioning as an intraoperative optical biopsy. Early feasibility studies by Sanai et al. and Martirosyan et al. established that CLE can reliably visualize cellular and vascular features of brain tumors intraoperatively, demonstrating high concordance with histopathology [5,15]. Furthermore, telepathology-based CLE interpretation has been shown to be feasible, suggesting that intraoperative decision-making could be supported by remote expert analysis [9]. These foundational studies were not metastasis-specific but were critical in showing that microscopic assessment can be integrated directly into surgical workflow.

**Figure 2:**
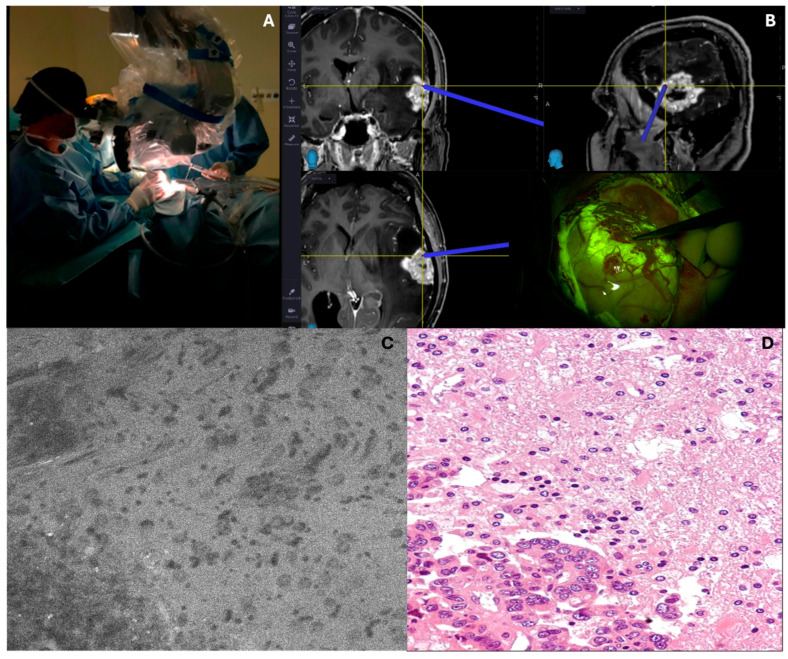
The CLE probe can be used freehand and placed directly in contact with the brain surface, provided that it is covered with its dedicated sterile sheath (**A**). (**B**) Preoperative imaging of a left temporal metastasis. The lower-right inset shows the site where the CLE probe was placed to perform the virtual biopsy. (**C**) Intraoperative CLE image acquired at the center of the brain lesion, showing a nest of tumor cells with hyperchromatic and irregular nuclei in the lower-left area, corresponding to the finding observed on definitive hematoxylin and eosin histology (**D**).

More recent studies using clinically integrated systems such as CONVIVO^®^ have extended these findings into practical intraoperative applications. Restelli et al. demonstrated that CLE can accurately identify pathological tissue at infiltration margins, reporting high diagnostic accuracy across CNS tumors, including metastases (Figure 2) [14]. Similarly, Wagner et al. showed that fluorescein-based CLE allows rapid intraoperative assessment compared with frozen section histology, although with somewhat lower specificity [8]. These findings suggest that CLE is best positioned as a complementary tool that provides rapid, spatially targeted information at the resection margin rather than replacing standard pathology.

Several studies have specifically emphasized the ability of CLE to interrogate different intraoperative zones. Höhne et al. demonstrated distinct CLE patterns between tumor core, tumor border, and perilesional tissue, supporting its use for intraoperative assessment of the MBPI [13]. Likewise, Belykh et al. reported that fluorescein-guided CLE allows visualization of tumor microstructure and architectural disruption in intraoperative samples, reinforcing its role in tissue characterization at the margin [10]. These observations underscore a fundamental limitation of conventional imaging: although MRI can suggest infiltrative behavior, it lacks the resolution to reliably detect microscopic tumor extension beyond the contrast-enhancing margin, a phenomenon consistently demonstrated in histopathological analyses of brain metastases [5].

An additional metastasis-specific aspect is highlighted by Brielmaier et al., who demonstrated that metastatic tumors exhibit significantly higher intracellular SF accumulation compared with primary brain tumors [12]. This finding is particularly relevant for margin assessment, as it suggests that metastatic tumor cells may be more readily identifiable on CLE, potentially improving detection of infiltrative disease at the interface. However, the same study also underscores technical limitations, including non-diagnostic images due to motion artifacts, hemorrhage, or suboptimal contrast, indicating that image quality and operator experience remain critical factors.

Taken together, the available evidence supports a consistent biological and technical interpretation. Brain metastases frequently exhibit infiltrative growth at the metastasis–brain interface, and CLE provides a feasible method for visualizing this interface intraoperatively. The ability to identify microscopic tumor infiltration in real time addresses a major limitation of current surgical practice, where margin assessment is otherwise indirect and delayed. In this context, CLE may contribute to a more biologically informed surgical strategy, including the potential application of supramarginal resection in selected cases.

Nevertheless, important limitations must be acknowledged. The current evidence base is characterized by relatively small cohorts, heterogeneous study designs, and frequent inclusion of mixed intracranial tumor populations, including gliomas, meningiomas, schwannomas, and metastatic lesions, with only a limited number of studies specifically designed to evaluate brain metastases and the metastasis–brain interface. Even in the most relevant studies, including the MetInfilt trial, the impact of CLE-guided margin assessment on clinically meaningful outcomes such as extent of resection, local recurrence, progression-free survival, or overall survival remains unproven. Consequently, the current literature supports the technical feasibility and biological plausibility of CLE more strongly than its demonstrated impact on oncological outcomes [5]. Furthermore, variability in specificity across studies highlights the ongoing challenge of distinguishing tumor infiltration from reactive or treatment-related changes at the periphery.

At present, CLE should be considered an adjunctive modality that may assist intraoperative decision-making rather than a stand-alone determinant of surgical strategy. Future prospective studies focusing specifically on brain metastases, with standardized imaging protocols and outcome measures, are required to define its role more precisely.

## 5. Conclusions

The current literature increasingly indicates that BM are often infiltrative beyond the radiological enhancing border. In this setting, CLE may provide additional microscopic information at the resection margin and could potentially support more biologically informed intraoperative decision-making; however, evidence demonstrating improvement in long-term oncological outcomes remains insufficient. Prospective studies specifically designed to test whether CLE-guided margin assessment improves local control are now needed to determine whether this promise can be translated into measurable clinical benefit.

## Figures and Tables

**Table 1:** **Characteristics of the studies included in the systematic review.** Summary of study design, patient population, CLE system, fluorescein usage, imaging modality, and reference standards across the included studies evaluating intraoperative confocal laser endomicroscopy for brain tumor surgery with emphasis on brain metastasis cases (BM).

Study	Year	Study Design	Patients (n)	BM (n)	CLE System	Fluorescein Use	Imaging Type	Reference Standard	Main Outcome
Abramov et al. [9]	2022	Feasibility study	11	1	CONVIVO^®^	Sodium fluorescein	In vivo	Histopathology	Remote interpretation of CLE images
Abramov et al. [11]	2023	Prospective feasibility study	30	3	CONVIVO^®^	Sodium fluorescein	In vivo	Frozen section and permanent histology	Diagnostic accuracy and feasibility
Belykh et al. [10]	2020	Prospective cohort	47	4	CLE	Sodium fluorescein	Ex vivo intraoperative imaging	Histopathology	Diagnostic performance of CLE
Brielmaier et al. [12]	2025	Experimental/clinical study	111	23	CLE	Sodium fluorescein	Ex vivo, in vivo	Histopathology	Fluorescein distribution patterns in metastases
Höhne et al. [13]	2021	Clinical experience study	12	5	CLE	Sodium fluorescein	In vivo	Histopathology	Visualization of tumor center, border and perilesional zones
Martirosyan et al. [7]	2016	Prospective cohort	74	1	Optiscan CLE	Sodium fluorescein	In vivo	Frozen and permanent histology	Diagnostic accuracy of CLE
Proescholdt et al. [5]	2025	Prospective clinical trial	50	50	CONVIVO^®^	Sodium fluorescein	In vivo	Histopathology	Characterization of metastasis–brain interface
Restelli et al. [14]	2025	Prospective clinical study	75	10	CONVIVO^®^	Sodium fluorescein	In vivo	Histopathology	CLE accuracy at infiltration margins
Sanai et al. [15]	2011	Feasibility study	33	2	Probe-based CLE	Sodium fluorescein	In vivo	Histopathology	Feasibility of intraoperative CLE imaging
Wagner et al. [8]	2024	Prospective multicenter trial	210	49	CONVIVO^®^	Sodium fluorescein	In vivo	Frozen section histology	Diagnostic accuracy compared with frozen section

## Data Availability

The raw data supporting the conclusions of this article will be made available by the authors on request.

## Supplementary Materials

The following supporting information can be downloaded at: https://www.mdpi.com/article/10.3390/jcm15124420/s1. Table S1: PRISMA 2020 Checklist; Table S2: Risk of bias assessment using the QUADAS-2 tool.

## References

[1] Sacks P., Rahman M. (2020). Epidemiology of Brain Metastases. Neurosurg. Clin. N. Am..

[2] Karschnia P., Le Rhun E., Vogelbaum M.A., Van Den Bent M., Grau S.J., Preusser M., Soffietti R., Von Baumgarten L., Westphal M., Weller M. (2021). The evolving role of neurosurgery for central nervous system metastases in the era of personalized cancer therapy. Eur. J. Cancer.

[3] Berghoff A.S., Rajky O., Winkler F., Bartsch R., Furtner J., Hainfellner J.A., Goodman S.L., Weller M., Schittenhelm J., Preusser M. (2013). Invasion patterns in brain metastases of solid cancers. Neuro-Oncology.

[4] Siam L., Bleckmann A., Chaung H.-N., Mohr A., Klemm F., Barrantes-Freer A., Blazquez R., Wolff H.A., Lüke F., Rohde V. (2015). The metastatic infiltration at the metastasis/brain parenchyma-interface is very heterogeneous and has a significant impact on survival in a prospective study. Oncotarget.

[5] Proescholdt M.A., Araceli T., Schebesch K.-M., Doenitz C., Wendl C., Evert K., Noeva E., Hoehne J., Riemenschneider M.J., Hirsch D. (2025). MetInfilt: A prospective trial highlighting the importance of the histological growth pattern in brain metastases. Transl. Oncol..

[6] Raore B., Schniederjan M., Prabhu R., Brat D.J., Shu H.-K., Olson J.J. (2011). Metastasis Infiltration: An Investigation of the Postoperative Brain–Tumor Interface. Int. J. Radiat. Oncol. Biol. Phys..

[7] Martirosyan N.L., Eschbacher J.M., Kalani M.Y.S., Turner J.D., Belykh E., Spetzler R.F., Nakaji P., Preul M.C. (2016). Prospective evaluation of the utility of intraoperative confocal laser endomicroscopy in patients with brain neoplasms using fluorescein sodium: Experience with 74 cases. Neurosurg. Focus.

[8] Wagner A., Brielmaier M.C., Kampf C., Baumgart L., Aftahy A.K., Meyer H.S., Kehl V., Höhne J., Schebesch K.-M., Schmidt N.O. (2024). Fluorescein-stained confocal laser endomicroscopy versus conventional frozen section for intraoperative histopathological assessment of intracranial tumors. Neuro-Oncology.

[9] Abramov I., Park M.T., Gooldy T.C., Xu Y., Lawton M.T., Little A.S., Porter R.W., Smith K.A., Eschbacher J.M., Preul M.C. (2022). Real-time intraoperative surgical telepathology using confocal laser endomicroscopy. Neurosurg. Focus.

[10] Belykh E., Zhao X., Ngo B., Farhadi D.S., Byvaltsev V.A., Eschbacher J.M., Nakaji P., Preul M.C. (2020). Intraoperative Confocal Laser Endomicroscopy Ex Vivo Examination of Tissue Microstructure During Fluorescence-Guided Brain Tumor Surgery. Front. Oncol..

[11] Abramov I., Park M.T., Belykh E., Dru A.B., Xu Y., Gooldy T.C., Scherschinski L., Farber S.H., Little A.S., Porter R.W. (2023). Intraoperative confocal laser endomicroscopy: Prospective in vivo feasibility study of a clinical-grade system for brain tumors. J. Neurosurg..

[12] Brielmaier M.C., Reifenrath J., Ganster F., Pensel N., Gempt J., Meyer B., Schlegel J., Wagner A. (2025). Fluorescein-distribution in confocal laser endomicroscopy allows for discrimination between primary brain tumours and metastases. Front. Surg..

[13] Höhne J., Schebesch K.-M., Zoubaa S., Proescholdt M., Riemenschneider M.J., Schmidt N.O. (2021). Intraoperative imaging of brain tumors with fluorescein: Confocal laser endomicroscopy in neurosurgery. Clinical and user experience. Neurosurg. Focus.

[14] Restelli F., Pollo B., Mazzapicchi E., Tramacere I., Broggi M., Falco J., Schiariti M., Stanziano M., Dimeco F., Ferroli P. (2025). Confocal endomicroscopy accuracy in identifying central nervous system tumors tissue at the infiltration margins: Results from a prospective clinical trial. J. Neurosurg. Sci..

[15] Sanai N., Eschbacher J., Hattendorf G., Coons S.W., Preul M.C., Smith K.A., Nakaji P., Spetzler R.F. (2011). Intraoperative Confocal Microscopy for Brain Tumors: A Feasibility Analysis in Humans. Oper. Neurosurg..

