# Supplementary material for: Confocal Laser Endomicroscopy in Brain Metastasis Surgery: A Systematic Review of the Evidence at the Tumor–Brain Interface
Source: J Clin Med. 2026 Jun 7;15(12):4420. doi: 10.3390/jcm15124420 (PMC13301885; doi:10.3390/jcm15124420)
Supplement: Supplementary file 1 [file jcm-15-04420-s001.zip › jcm-4308576-supplementary Table S2.pdf]

Study	Patient Selection	Index Test (CLE)	Reference Standard (Histology)	Flow and Timing	Applicability Concerns	Overall Risk of Bias
**Sanai et al., 2011**	Unclear	Low	Low	Unclear	Moderate	Moderate
**Martirosyan et al., 2016**	Low	Low	Low	Low	Moderate	Low
**Belykh et al., 2020**	Low	Low	Low	Low	Low	Low
**Höhne et al., 2021**	Unclear	Low	Low	Unclear	Moderate	Moderate
**Abramov et al., 2022**	Unclear	Low	Low	Unclear	Moderate	Moderate
**Abramov et al., 2023**	Low	Low	Low	Low	Low	Low
**Wagner et al., 2024**	Low	Low	Low	Low	Low	Low
**Proescholdt et al., 2025**	Low	Low	Low	Low	Low	Low
**Restelli et al., 2025**	Low	Low	Low	Low	Low	Low
**Brielmaier et al., 2025**	Unclear	Low	Low	Unclear	Moderate	Moderate

Supplementary Table S2. Risk of bias assessment using the QUADAS-2 tool. The methodological quality of included studies was evaluated using the Quality Assessment of Diagnostic Accuracy Studies-2 (QUADAS-2) framework. Each study was assessed across four domains: patient selection, index test (confocal laser endomicroscopy), reference standard (histopathology), and flow and timing. Risk of bias was categorized as low, high, or unclear. Applicability concerns reflect the relevance of each study to the present systematic review focusing on intraoperative confocal laser endomicroscopy using sodium fluorescein in brain metastasis surgery.
